# Genetic and genomic analysis of hyperlipidemia, obesity and diabetes using (C57BL/6J × TALLYHO/JngJ) F2 mice

**DOI:** 10.1186/1471-2164-11-713

**Published:** 2010-12-19

**Authors:** Taryn P Stewart, Hyoung Yon Kim, Arnold M Saxton, Jung Han Kim

**Affiliations:** 1 Department of Pharmacology, Physiology and Toxicology, Joan C. Edwards School of Medicine, Marshall University, Huntington, WV 25755, USA; 2 Department of Nutrition, The University of Tennessee, Knoxville, TN 37996, USA; 3 Department of Animal Science, The University of Tennessee, Knoxville, TN 37996, USA

## Abstract

**Background:**

Type 2 diabetes (T2D) is the most common form of diabetes in humans and is closely associated with dyslipidemia and obesity that magnifies the mortality and morbidity related to T2D. The genetic contribution to human T2D and related metabolic disorders is evident, and mostly follows polygenic inheritance. The TALLYHO/JngJ (TH) mice are a polygenic model for T2D characterized by obesity, hyperinsulinemia, impaired glucose uptake and tolerance, hyperlipidemia, and hyperglycemia.

**Results:**

In order to determine the genetic factors that contribute to these T2D related characteristics in TH mice, we interbred TH mice with C57BL/6J (B6) mice. The parental, F1, and F2 mice were phenotyped at 8, 12, 16, 20, and 24 weeks of age for 4-hour fasting plasma triglyceride, cholesterol, insulin, and glucose levels and body, fat pad and carcass weights. The F2 mice were genotyped genome-wide and used for quantitative trait locus (QTL) mapping. We also applied a genetical genomic approach using a subset of the F2 mice to seek candidate genes underlying the QTLs. Major QTLs were detected on chromosomes (Chrs) 1, 11, 4, and 8 for hypertriglyceridemia, 1 and 3 for hypercholesterolemia, 4 for hyperglycemia, 11 and 1 for body weight, 1 for fat pad weight, and 11 and 14 for carcass weight. Most alleles, except for Chr 3 and 14 QTLs, increased phenotypic values when contributed by the TH strain. Fourteen pairs of interacting loci were detected, none of which overlapped the major QTLs. The QTL interval linked to hypercholesterolemia and hypertriglyceridemia on distal Chr 1 contains *Apoa2 *gene. Sequencing analysis revealed polymorphisms of *Apoa2 *in TH mice, suggesting *Apoa2 *as the candidate gene for the hyperlipidemia QTL. Gene expression analysis added novel information and aided in selection of candidates underlying the QTLs.

**Conclusions:**

We identified several genetic loci that affect the quantitative variations of plasma lipid and glucose levels and obesity traits in a TH × B6 intercross. Polymorphisms in *Apoa2 *gene are suggested to be responsible for the Chr 1 QTL linked to hypercholesterolemia and hypertriglyceridemia. Further, genetical genomic analysis led to potential candidate genes for the QTLs.

## Background

Diabetes is one of the most devastating and prevalent diseases in humans. According to data from the 2007 National Diabetes Fact Sheet, 23.6 million people in the United States (7.8% of the population) have diabetes http://www.diabetes.org/diabetes-basics/diabetes-statistics/. Type 2 diabetes (T2D) is the most common form of diabetes and is often associated with a collection of metabolic disorders including dyslipidemia and obesity which substantially magnifies the mortality and morbidity related to T2D [1,2]. Hypertriglyceridemia is the main lipid abnormality in T2D patients that usually occurs years before diabetes, and is a strong predictor of diabetes [2]. The genetic contribution to human T2D and related metabolic disorders is evident, and mostly follows polygenic inheritance [3,4].

TALLYHO/JngJ (TH) mice are a polygenic model for T2D characterized by glucose intolerance and hyperglycemia (limited to males) and show metabolic abnormalities including obesity, insulin resistance, hyperinsulinemia, and hyperlipidemia [5-7]. The TH male mice exhibit a striking rise in plasma triglyceride levels at an early age when their plasma glucose levels are steadily rising [6]. On the other hand, TH female mice do not exhibit this triglyceride spike although they maintain hypertriglyceridemia compared with age- and sex-matched C57BL/6J (B6) mice. Some degree of vascular dysfunction is also reported in TH mice [8,9].

In the present study, we performed a genome-wide scan to search for quantitative trait loci (QTLs) affecting hypertriglyceridemia in TH mice using male F2 mice from a cross of B6 × TH. We also searched for loci linked to hypercholesterolemia, hyperinsulinemia, obesity, and hyperglycemia. Finally, we applied a genetical genomic approach and assessed the data systemically for candidate genes at the QTLs.

## Results

### Phenotypes in parental, F1, and F2 mice

Body weights and 4-hour fasting plasma levels of triglyceride, total cholesterol, insulin, and glucose were measured at 8, 12, 16, 20, and 24 weeks of age. At 24 weeks of age, mice were killed and the five regional fat pads were dissected and weighed. In the F2 intercross mice, where B6 and TH genomes are mixed and reassembled, there was wide variation in trait values that even exceeded the ranges in parental mice (Table 1). This indicates that multiple genetic variants between the parental strains were responsible for the expression of traits.

**Table 1:** Phenotypes of the parental, F1, and F2 mice (males).

	B6 (n)	F1 (n)	TH (n)	F2 (n) (lowest, highest)	16 F2 mice chosen for microarray Lower (n = 8); Upper (n = 8)
Triglyceride (mg/dl)					
8 wk	63 ± 12 (9)^a^	153 ± 12 (15)^b^	438 ± 41 (7)^c^	168 ± 3 (368) (60, 472)	130 ± 14; 207 ± 22 (P = 0.009)
12 wk	68 ± 8 (14)^a^	186 ± 11 (19)^b^	237 ± 53 (9)^b^	166 ± 3 (382) (44, 357)	135 ± 14; 230 ± 22 (P = 0.003)
16 wk	63 ± 3 (18)^a^	168 ± 8 (19)^b^	438 ± 39 (16)^c^	167 ± 4 (384) (20, 486)	120 ± 15; 191 ± 27 (P = 0.038)
20 wk	71 ± 7 (18)^a^	180 ± 20 (19)^b^	367 ± 32 (16)^c^	181 ± 4 (377) (56, 479)	126 ± 17; 336 ± 24 (P < 0.0001)
24 wk	60 ± 6 (18)^a^	221 ± 19 (19)^b^	368 ± 33 (16)^c^	181 ± 4 (375) (40, 436)	81 ± 7; 331 ± 17 (P < 0.0001)

Cholesterol (mmol/l)					
8 wk	2.9 ± 0.2 (9)^a^	2.3 ± 0.1 (15)^b^	4.0 ± 0.1 (7)^c^	2.9 ± 0.1 (365) (0.7, 7.6)	2.2 ± 0.2; 2.9 ± 0.4 (P = 0.09)
12 wk	2.6 ± 0.2 (14)^a^	3.1 ± 0.2 (19)^b^	2.9 ± 0.1 (9)^b^	3.1 ± 0.1 (378) (0.4, 8.5)	2.5 ± 0.3; 3.1 ± 0.5 (P = 0.36)
16 wk	3.0 ± 0.3 (19)^a^	2.6 ± 0.1 (19)^a^	3.6 ± 0.3 (16)^b^	3.0 ± 0.1 (384) (1.0, 6.8)	2.5 ± 0.3; 3.6 ± 0.4 (P = 0.04)
20 wk	2.1 ± 0.1 (18)^a^	3.5 ± 0.3 (19)^b^	4.7 ± 0.3 (16)^c^	3.3 ± 0.1 (377) (1.1, 6.8)	2.7 ± 0.3; 3.6 ± 0.2 (P = 0.03)
24 wk	1.9 ± 0.1 (18)^a^	3.6 ± 0.1 (19)^b^	4.0 ± 0.4 (16)^b^	3.3 ± 0.1 (375) (1.3, 8.1)	2.8 ± 0.2; 3.1 ± 0.5 (P = 0.58)

Glucose (mg/dl)					
8 wk	175 ± 10 (9)^a^	175 ± 11 (15)^a^	292 ± 48 (7)^b^	176 ± 2 (368) (98, 353)	163 ± 11; 183 ± 9 (P = 0.20)
12 wk	132 ± 6 (14)^a^	147 ± 9 (19)^a^	252 ± 40 (9)^b^	166 ± 2 (382) (45, 473)	145 ± 8; 168 ± 11 (P = 0.10)
16 wk	181 ± 14 (19)^a^	166 ± 4 (19)^a^	460 ± 38 (16)^b^	169 ± 3 (384) (62, 575)	144 ± 15; 160 ± 11 (P = 0.40)
20 wk	177 ± 10 (18)^a^	156 ± 5 (19)^a^	364 ± 36 (16)^b^	165 ± 3 (378) (64, 492)	144 ± 17; 163 ± 13 (P = 0.40)
24 wk	175 ± 10 (18)^a^	162 ± 8 (19)^a^	387 ± 33 (16)^b^	162 ± 3 (375) (40, 599)	131 ± 9; 128 ± 13 (P = 0.83)

Insulin (ng/ml)					
8 wk	0.07 ± 0.02 (9)^a^	0.96 ± 0.17 (15)^b^	1.81 ± 0.41 (7)^c^	1.31 ± 0.06 (384) (0.01, 9.71)	1.45 ± 0.31; 1.36 ± 0.28 (P = 0.83)
12 wk	0.22 ± 0.07 (13)^a^	1.12 ± 0.15 (19)^b^	0.98 ± 0.33 (9)^b^	1.55 ± 0.08 (376) (0.06, 12.36)	0.80 ± 0.14; 1.28 ± 0.17 (P = 0.05)
16 wk	0.35 ± 0.09 (18)^a^	1.24 ± 0.13 (19)^b^	0.98 ± 0.26 (15)^b^	2.17 ± 0.11 (381) (0.07, 15.03)	1.19 ± 0.23; 1.94 ± 0.43 (P = 0.20)
20 wk	0.42 ± 0.11 (18)^a^	2.14 ± 0.26 (19)^b^	1.56 ± 0.41 (16)^b^	2.39 ± 0.15 (376) (0.02, 23.18)	1.12 ± 0.36; 2.35 ± 0.47 (P = 0.06)
24 wk	0.41 ± 0.10 (10)^a^	2.78 ± 0.55 (16)^b^	2.31 ± 0.56 (13)^b^	2.70 ± 0.16 (371) (0.06, 23.97)	2.59 ± 1.10; 5.13 ± 2.10 (P = 0.30)

Body weight (g)					
8 wk	22 ± 0.4 (14)^a^	30 ± 0.5 (15)^b^	31 ± 0.5 (7)^b^	29 ± 0.2 (385) (20, 40)	30 ± 1.1; 29 ± 0.9 (P = 0.33)
12 wk	24 ± 0.5 (18)^a^	33 ± 0.6 (19)^b^	32 ± 0.6 (16)^b^	33 ± 0.2 (385) (23, 45)	33 ± 1.2; 33 ± 1.2 (P = 0.65)
16 wk	25 ± 0.5 (18)^a^	36 ± 0.7 (19)^b^	34 ± 0.8 (16)^b^	35 ± 0.2 (383) (24, 49)	35 ± 1.5; 36 ± 1.3 (P = 0.66)
20 wk	27 ± 0.5 (18)^a^	38 ± 0.7 (19)^b^	36 ± 1.0 (16)^c^	37 ± 0.3 (378) (25, 52)	36 ± 2.0; 38 ± 1.1 (P = 0.39)
24 wk	28 ± 0.4 (18)^a^	41 ± 0.6 (19)^b^	36 ± 1.4 (16)^c^	39 ± 0.3 (373) (25, 59)	38 ± 2.2; 41 ± 1.3 (P = 0.26)

Fat pad & Carcass weights (g)					
IG	0.34 ± 0.02 (18)^a^	1.37 ± 0.07 (19)^b^	0.69 ± 0.15 (16)^c^	0.99 ± 0.03 (372) (0.07, 2.66)	0.64 ± 0.14; 1.14 ± 0.10 (P = 0.01)
ED	0.43 ± 0.03 (18)^a^	1.86 ± 0.07 (19)^b^	0.98 ± 0.23 (16)^c^	1.41 ± 0.03 (372) (0.19, 3.21)	1.11 ± 0.25; 1.69 ± 0.19 (P = 0.08)
MS	0.11 ± 0.01 (18)^a^	0.60 ± 0.03 (19)^b^	0.24 ± 0.06 (16)^c^	0.40 ± 0.01 (372) (0.07, 1.30)	0.35 ± 0.12; 0.46 ± 0.04 (P = 0.40)
RP	0.12 ± 0.01 (18)^a^	0.64 ± 0.02 (19)^b^	0.26 ± 0.06 (16)^c^	0.43 ± 0.01 (371) (0.05, 1.47)	0.31 ± 0.07; 0.63 ± 0.13 (P = 0.05)
SC	0.14 ± 0.01 (18)^a^	0.72 ± 0.04 (19)^b^	0.32 ± 0.09 (16)^c^	0.50 ± 0.02 (372) (0.05, 3.26)	0.32 ± 0.08; 0.59 ± 0.06 (P = 0.01)
Sum	1.14 ± 0.06 (18)^a^	5.17 ± 0.20 (19)^b^	2.34 ± 0.55 (16)^c^	3.72 ± 0.09 (371) (0.52, 8.59)	2.69 ± 0.61; 4.51 ± 0.31 (P = 0.02)
Carcass	26 ± 0.4 (18)^a^	34 ± 0.5 (19)^b^	31 ± 1.0 (16)^c^	33 ± 0.2 (371) (23, 47)	34 ± 1.8; 34 ± 1.0 (P = 0.91)

Data are presented as mean ± sem. Means labeled with different letters are significantly different from one another comparing B6, F1 and TH (P < 0.05). wk, week; IG, inguinal; ED, epididymal; MS, mesenteric; RP, retroperitoneal including perirenal; SC, subscapular; Sum, sum of the 5 fat pads above; B6, C57BL/6J; TH, TALLYHO/JngJ.

Hypertriglyceridemia appeared to be inherited in a semidominant manner since the mean plasma level of triglyceride of F1 (B6 × TH) progeny was intermediate between the means of the two parental strains (Table 1). Hypercholesterolemia, on the other hand, showed a complex inheritance.

The F1 population showed a mean plasma glucose level that was indistinguishable from B6 strain, suggesting that hyperglycemia is inherited in a recessive manner for the TH alleles (Table 1). Plasma insulin levels in F1 mice were between the parental values at 8 weeks of age, but became similar to TH mice at later ages.

The F1 mice initially showed higher body weights than B6 mice, but comparable to TH mice (Table 1). However, at later ages hybrid vigor (or heterosis), where a first-generation hybrid displays superior phenotypic expression over their parents, was observed for body weights. Increases both in fat mass (measured by fat pad weights) and lean mass (measured by carcass weight) appeared to contribute to the increased body weights in the F1 mice.

### Significant QTLs identified by composite interval genome-wide scans

**Figure 1:**
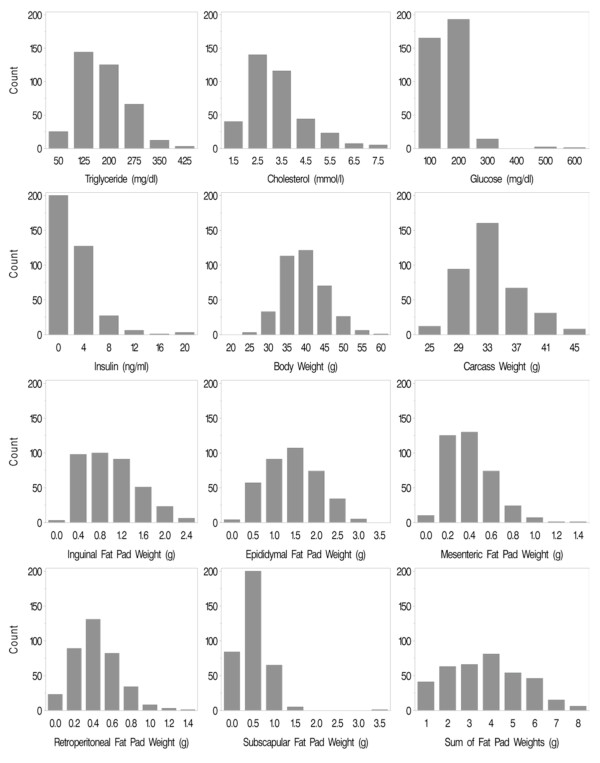
**Histogram showing distribution of traits at 24 weeks of age in F2 mice**. We interbred TH mice with B6 mice, and the resultant F2 mice (male) were phenotyped at 8, 12, 16, 20, and 24 weeks of age for 4-hour fasting plasma triglyceride, total cholesterol, insulin, and glucose levels and body, fat pad and carcass weights. Count is the number of mice.

**Figure 2:**
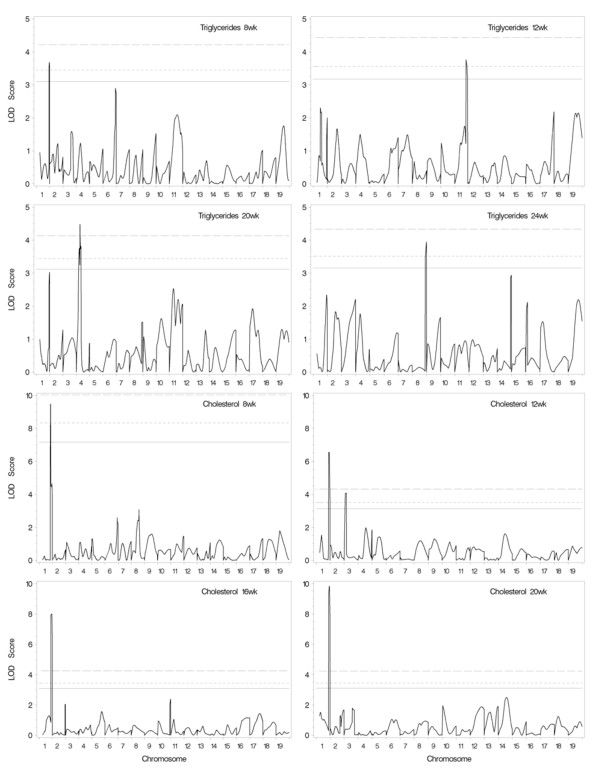
**Plot of one-dimensional genome-wide scans for (B6 × TH) F2 male progeny on 19 autosomes**. The associated phenotypic traits are plasma triglyceride and cholesterol levels as indicated. The lod score is plotted as a function of genome location. The horizontal lines represent critical values at the 99% (*P *< 0.01), 95% (*P *< 0.05) and 90% (*P *< 0.1) significance levels.

**Figure 3:**
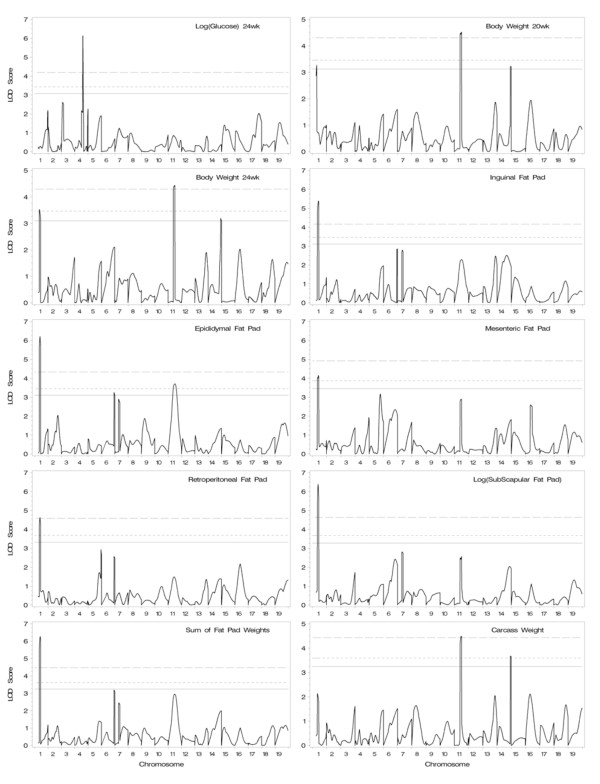
**Plot of one-dimensional genome-wide scans for (B6 × TH) F2 male progeny on 19 autosomes**. The associated phenotypic traits are plasma glucose levels and body, fat pad and carcass weights as indicated. The lod score is plotted as a function of genome location. The horizontal lines represent critical values at the 99% (*P *< 0.01), 95% (*P *< 0.05) and 90% (*P *< 0.1) significance levels.

We collected approximately 380 F2 male mice, and individual mice were genotyped with 68 simple sequence length polymorphism (SSLP) markers at approximately 20-cM intervals. A genome-wide QTL analysis was then performed for the traits in the male F2 population at 8, 12, 16, 20, and 24 weeks of age. Figure 1 represents the distribution of trait values at 24 weeks of age, with most traits showing approximately normal distributions, but a few traits had positive skew. Similar distribution patterns were observed at all other ages for each trait (not shown). QTLs with genome-wide significance levels > 5% are summarized in Table 2. Lod score plots of genome-wide scans are depicted in Figure 2 and 3 for traits with significant QTL.

**Table 2:** Summary of major QTLs detected in the F2 mice.

	Chr	Best location, cM (CI)	Closest marker to peak LOD	Peak LOD	**GW Sig**.	%		Phenotype value	
							**B6/B6 (n)**	**B6/TH (n)**	**TH/TH (n)**

Triglyceride (mg/dl)									
8 wk	1	95.7 (25.7, 96.9)	*D1Mit113*	3.67	S	3.63	157 ± 7 (89)^a^	164 ± 4 (201)^a^	188 ± 6 (78)^b^
12 wk	11	65 (20, 79)	*D11Mit132*	3.76	S	1.72	153 ± 5 (90)^a^	169 ± 4 (182)^b^	173 ± 6 (109)^b^
20 wk	4	31.3 (21.3, 41.3)	*D4Mit178*	4.47	VS	4.82	161 ± 6 (92)^a^	178 ± 5 (201)^a^	210 ± 9 (83)^b^
24 wk	8	55.75 (50, 55.7)	*D8Mit242*	3.94	S	4.29	159 ± 7 (97)^a^	185 ± 5 (186)^b^	199 ± 8 (91)^b^

Cholesterol (mmol/l)									
8 wk	1	86.7 (84.7, 87.7)	*D1Mit113*	11.83	VS	4.96	2.5 ± 0.1 (88)^a^	2.9 ± 0.1 (200)^b^	3.2 ± 0.1 (77)^c^
12 wk	1	89.7 (86.7, 96.7)	*D1Mit113*	6.55	VS	6.76	2.6 ± 0.1 (88)^a^	3.2 ± 0.1 (211)^b^	3.5 ± 0.1 (79)^c^
	3	10.6 (5.6, 11.6)	*D3Mit304*	4.07	S	4.31	3.5 ± 0.1 (102)^a^	3.0 ± 0.1 (177)^b^	2.9 ± 0.1 (99)^b^
16 wk	1	93.7 (86.7, 96.9)	*D1Mit113*	7.99	VS	8.62	2.6 ± 0.1 (92)^a^	3.0 ± 0.1 (212)^b^	3.5 ± 0.1 (80)^c^
20 wk	1	92.7 (86.7, 96.9)	*D1Mit113*	9.83	VS	11.05	2.8 ± 0.1 (89)^a^	3.2 ± 0.1 (209)^b^	3.9 ± 0.1 (79)^c^

Glucose (mg/dl)									
24 wk	4	67.3 (66.3, 70.3)	*D4Mit312*	6.13	VS	2.89	147 ± 4 (102)^a^	167 ± 4 (205)^b^	172 ± 8 (66)^b^

Body weight (g)									
20 wk	11	41 (32, 43)	*D11Mit41*	4.51	VS	2.17	36 ± 0.5 (72)^a^	38 ± 0.4 (199)^b^	38 ± 0.5 (103)^b^
24 wk	11	41 (32, 43)	*D11Mit41*	4.43	VS	1.74	38 ± 0.6 (72)^a^	40 ± 0.4 (197)^b^	40 ± 0.6 (103)^b^
	1	35.7 (33.7, 44.7)	*D1Mit215*	3.52	S	4.15	37 ± 0.6 (92)^a^	40 ± 0.4 (180)^b^	41 ± 0.6 (101)^b^

Fat pad weight (g)									
IG	1	41.7 (33.7, 44.7)	*D1Mit215*	5.38	VS	5.88	0.79 ± 0.05 (92)^a^	1.02 ± 0.04 (179)^b^	1.12 ± 0.05 (101)^b^
ED	1	39 (33.7, 44.7)	*D1Mit215*	6.20	VS	7.02	1.15 ± 0.06 (92)^a^	1.43 ± 0.05 (179)^b^	1.61 ± 0.06 (101)^c^
MS	1	41.7 (33.7, 44.7)	*D1Mit215*	4.14	S	4.78	0.32 ± 0.02 (92)^a^	0.42 ± 0.02 (179)^b^	0.43 ± 0.02 (101)^b^
RP	1	39 (33.7, 44.7)	*D1Mit215*	4.62	VS	5.40	0.33 ± 0.02 (91)^a^	0.44 ± 0.02 (179)^b^	0.47 ± 0.02 (101)^b^
SC	1	39 (33.7, 44.7)	*D1Mit215*	6.39	VS	7.24	0.37 ± 0.03 (92)^a^	0.54 ± 0.03 (179)^b^	0.56 ± 0.03 (101)^b^
Sum	1	40.7 (33.7, 44.7)	*D1Mit215*	6.26	VS	7.01	2.9 ± 0.16 (92)^a^	3.8 ± 0.13 (179)^b^	4.2 ± 0.18 (101)^b^

Carcass weight (g)									
	11	40 (32, 43)	*D11Mit41*	4.48	VS	1.88	32 ± 0.4 (72)^a^	34 ± 0.3 (197)^b^	34 ± 0.4 (102)^b^
	14	72.5 (65.5, 76.1)	*D14Mit107*	3.67	S	4.53	34 ± 0.5 (81)^a^	34 ± 0.3 (193)^a^	32 ± 0.4 (96)^b^

Phenotypic data are presented as mean ± sem. Means labeled with different letters are significantly different from one another (P < 0.05). Chr, chromosome; CI, 2 LOD support interval; %, total variance explained by R-square; IG, inguinal; ED, epididymal; MS, mesenteric; RP, retroperitoneal including perirenal; SC, subscapular; Sum, sum of the 5 fat pads above; GW Sig., genome-wide significance; S, significant; VS, very significant.

#### Triglyceride

Four significant QTLs for plasma triglyceride level had age-specific activity; loci near *D1Mit113 *on chromosome (Chr) 1 for 8 week, *D11Mit132 *on Chr 11 for 12 week, *D4Mit178 *on Chr 4 for 20 week, and *D8Mit242 *on Chr 8 for 24 week. Hypertriglyceridemic contributions were from TH genome. TH alleles for Chr 1 and Chr 4 QTLs appeared to be recessive while Chr 11 and Chr 8 QTLs were dominant.

#### Cholesterol

A very significant QTL near *D1Mit113 *on Chr 1 was linked to plasma total cholesterol levels. The hypercholesterolemic contribution of the locus was from the TH genome and appeared to be additive for all ages. In addition, a QTL near *D3Mit304 *on proximal Chr 3 was also responsible for the plasma total cholesterol levels at 12 week; the B6 allele at this locus was associated with increased plasma levels in total cholesterol, appearing recessive.

#### Glucose

On distal Chr 4, a QTL near *D4Mit312 *was very significantly linked to the plasma glucose levels, with 2 LOD support interval (CI) of 66.3 - 70.3 cM. For that locus, the TH alleles were associated with increased plasma glucose levels, and inheritance appeared to be dominant for the TH allele.

#### Insulin

No significant linkages were observed for plasma insulin levels.

#### Body weight

Two significant QTLs linked to body weight were identified near *D11Mit41 *on Chr 11 and *D1Mit215 *on Chr 1. For both QTLs, the TH alleles contributed to increased body weights with dominant action.

#### Fat pad weight

A significant QTL linked to fat pad weights was identified near *D1Mit215 *on Chr 1. The TH allele was associated with increases in all regional fat pad weights studied and appeared dominant for all except epididymal fat pad weights.

#### Carcass weight

Two QTLs near *D11Mit41 *on Chr 11 and *D14Mit107 *on Chr 14, respectively, were significantly linked to carcass weights (surrogate lean body mass). The TH alleles and the B6 alleles were associated with higher carcass weight at Chr 11 locus and Chr 14 locus, respectively. The Chr 11 QTL location overlapped the QTL associated with body weight.

### Epistasis and interacting QTLs

A pair-wise genome scan was conducted to examine all marker-marker interactions and identified 14 significant epistatic interacting QTLs; 4 for triglyceride, 5 for total cholesterol, 3 for glucose, and 2 for fat pad weight (Table 3). When we compared these epistatic QTLs with the single QTLs identified by single-locus genome scans, no locations overlapped.

**Table 3:** Summary of all QTL pairs detected using pair-wise scans in the F2 mice.

	QTL 1			QTL 2			LOD			%
**Trait**	**Chr**	**Best location (cM)**	**Closest marker to peak LOD**	**Chr**	**Best location (cM)**	**Closest marker to peak LOD**	**Full**	**Add**	**Int**	

Glu 12 wk	15	53.7	*D15Mit2*	19	27	*D19Mit30*	10.4	3.6	6.8	8.5
	5	36	*D5Mit80*	7	28.7	*D7Mit231*	9.3	2.9	6.4	6.5
	12	53	*D12Mit233*	19	28	*D19Mit30*	9.3	2.8	6.5	5.8

Tg 16 wk	3	21.6	*D3Mit304*	13	38	*D13Mit26*	12.3	2.1	10.2	4.8

Tg 20 wk	7	79.7	*D7Mit109*	14	59.5	*D14Mit102*	11.7	1.3	10.4	4.8
	10	24	*D10Mit130*	17	75	*D17Mit123*	10.8	2.8	8.1	5.0
	5	99	*D5Mit101*	16	51.3	*D16Mit152*	11.1	0.9	10.2	3.1

Chol 8 wk	5	33	*D5Mit80*	17	30	*D17Mit54*	12.9	2.1	10.8	4.8
	5	107	*D5Mit101*	14	57.5	*D14Mit107*	9.3	1.9	7.4	4.0

Chol 24 wk	1	68.7	*D1Mit26*	10	50	*D10Mit11*	13.4	5.8	7.6	7.5
	15	45.7	*D5Mit2*	16	57.3	*D16Mit152*	10.5	2.0	8.6	4.2
	4	14.3	*D4Mit97*	5	98	*D5Mit101*	13.6	1.8	11.8	3.7

IG FPW	7	82.7	*D7Mit109*	8	52	*D8Mit242*	9.9	3.0	6.9	3.7

Sum FPW	7	71.7	*D7Mit109*	17	76	*D17Mit123*	9.8	2.7	7.1	3.9

Chr, chromosome; Full, the Lod score for the full model (including additive effects and interaction); Add, the Lod score for two locus additive effects; Int, the Lod score for the interaction (Full - Add); %, total variance explained by R-square; Glu, glucose; Tg, trighlyceride; Chol, cholesterol; IG FPW, inguinal fat pad weight; Sum FPW, sum of the 5 regional fat pad weights; wk, week.

**Figure 4:**
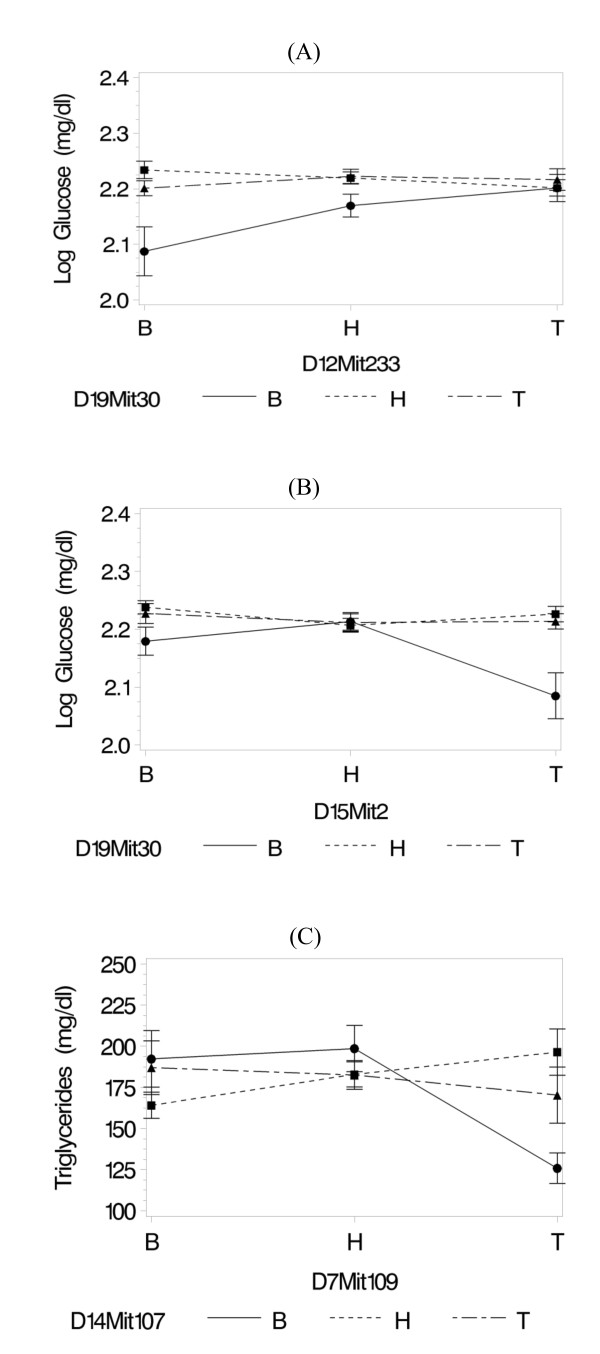
**Two locus interactive effects in (B6 × TH) F2 male progeny**. The associated phenotypic traits are hyperglycemia (A and B) and hypertriglyceridemia (C). Lines connect means ± SEM of the plasma glucose or triglyceride levels for one marker on the X axis as homozygous for B6 (B), heterozygous for TH and B6 (H) or homozygous for TH (T) associated with another marker homozygous for B6 (B, solid line), heterozygous for B6 and TH (H, dotted line) or homozygous for TH (T, dashed line).

It was noteworthy that a locus near *D19Mit30 *on Chr 19 interacted with two different loci affecting plasma glucose levels, one near *D15Mit2 *on Chr 15 and the other near *D12Mit233 *on Chr 12. At 12 weeks of age, mice that were homozygous for the B6 alleles at the *D19Mit30 *locus exhibited a significantly lower plasma glucose level when they were homozygous either for the TH allele at the *D15Mit2 *locus or for the B6 allele at the *D12Mit233 *locus as compared with other possible genotype combinations at the loci (Figure 4A and 4B).

Similarly, mice that were homozygous for the B6 allele at the *D14Mit107 *locus on Chr 14 had a significantly lower plasma triglyceride level if they were homozygous for the TH alleles at the *D7Mit109 *locus on Chr 7 (20 weeks of age) (Figure 4C).

### Candidate genes using genetical genomics in the F2 mice

In order to seek candidate genes underlying the QTLs, we applied a genetical genomics approach using a subset of the F2 mice (n = 16) with differential hypertriglyceridemia, but not overtly diabetic. We analyzed gene expression levels in four critical tissues associated with diabetes, including liver, adipose tissue, skeletal muscle, and pancreas. ANOVA was used to identify associations between markers and gene expression in each tissue. In total, 8764 gene expression traits in liver, 1410 in adipose tissue, 1832 in skeletal muscle, and 4130 in pancreas exhibited an association with the markers tested.

Among these, we searched for gene expression traits that were associated with the markers linked to physiological traits to select candidate genes for the QTLs (Table 4). When *cis*-acting transcript is defined as a locus residing within 20 cM of the gene location [10], this analysis revealed 4 putative *cis*-acting transcripts; coiled-coil domain containing 46 (*Ccdc46*) gene in liver for the hypertriglyceridemia QTL near *D11Mit132*, signal-regulatory protein beta 1 (*Sirpb1*) gene in adipose tissue for the hypercholesterolemia QTL near *D3Mit304*, RIKEN cDNA 1700009P17 (*1700009P17Rik*) gene in liver for the hypercholesterolemia QTL near *D1Mit113*, and chymotrypsin C (caldecrin) (*Ctrc*) gene in pancreas for the diabetes QTL near *D4Mit312*. These 4 genes have mostly unknown association with lipid and glucose metabolism or obesity.

**Table 4:** Gene expression associated with physiological trait QTL markers in the subset of F2 mice (n = 16).

Marker	Tissue	Gene
*D1Mit113*	Adipose tissue	*Abcb9, Dub2a, Myo1a, 4930571K23Rik, Pnma2, Tcea2, Fahd2a, Zmiz1, Actr1b, Rab3 d, Zscan2, Myo10, Arhgap8, Mrgpra2, 4933431C10Rik, Oprm1*
	Liver	*Clasp1, **1700009P17Rik**, 9130009M17Rik, Wdr40b, Rasal2, Gabra3, Mrgpre, Lsm2, 2610039C10Rik, Zfp354b*
	Muscle	*A630033E08Rik, Pcdhgc4, Kbtbd3, Tbc1d23, Tmem181*
	Pancreas	*Snca, Lrch1, Plcl1, Lpin2, 2410005O16Rik, Abcb9, Rpp30, Sepw1*

*D11Mit132*	Adipose tissue	*Rab33a, Homer1, Cntfr, 2310068J16Rik, 4930427A07Rik*
	Liver	*Diablo, Snx29, Galnt3, H13, Dqx1, 4930563E22Rik, 4931429I11Rik, Tmem129, Rasgrp1, 4930452B06Rik, 4930528G23Rik, 4933413J09Rik, **Ccdc46**, Zfp61, Ypel2, Hs3st3a1, 4933404O12Rik, Lgi3, Cplx2, Pif1, Fkrp, 9530010C24Rik, Rasa4, Rlbp1, Hoxa10, Lysmd2, Mipol1, Hps1, Csl, Fgf18, Mcpt9, Rnf151, Ggt7, Sema6c, 4921511C04Rik, Med13l, 4930432H15Rik, A330021E22Rik, Vwc2, Nfasc, Zic4, Htt, A230001M10Rik, Zic2, Cnnm4, Eif4e2, Tbx19, Kcnab3, Napg*
	Muscle	*Tmem144, Dsc3, Tm9sf1, Fancb, Eif3 d, Gtf2e2, Tph1, 2900022B07Rik, 2010004M13Rik, Mgat3*
	Pancreas	*Ednrb, Epn2, Rnf214, Spata17, 2310047O13Rik, Dnajb4, 4930455H04Rik, Emp2, Cds2, Slc2a13, Cybb, 3110052M02Rik, 4933403F05Rik, Skiv2l2, Klhl13, Myo1b, Supt16 h, Usp9x, Crot, Ogt, P4ha1, Igf1r, Akt1s1, Rabepk, Ptprj, Pml, Chd1, Tspan8, 6430531B16Rik, 4933411K20Rik, Tpst1, Bnip2, Map4k4, Nup50, Fzd8, Olfml2b, Anapc4, Car8, Eif3s10*

*D4Mit178*	Adipose tissue	*Trpv4, Azgp1, Klf1, B3galt6, Cgref1, Ptprk, 1110033F14Rik, Gpm6b*
	Liver	*Tcfe2a, Wipi1, Lyrm2, Nudt16l1, Ttf2, Gngt2, 9430051O21Rik, Cd86, Rad23b, Tmem175, Pola1, Ankrd49*
	Pancreas	*Lmbr1, 4932415G12Rik, Clock, Itm2a, Tcf25*

*D8Mit242*	Adipose tissue	*Adrb3, Isca1, Cep55, Ppp1r9a*
	Liver	*6530406A20Rik, Lsm6*

*D3Mit304*	Adipose tissue	*Rhod, Akt3, Dnmt3a, Grhl1, Dppa3, Rpl7, Krt20, Tm7sf4, Palb2, Hk3, Dusp16, Acad9, Pctk3, Cxcl2, Fabp7, Top3b, Slamf6, **Sirpb1***
	Liver	*Exosc9, Kcne3, Acad9, 2610202C22Rik, Dmc1, Mapk8*
	Muscle	*Os9, Coro1c, Gna-rs1, 4921508A21Rik, 5830420C07Rik, H2-T18*
	Pancreas	*4632419K20Rik, Rbbp8, Foxk2, Ccdc7, Stag1, Uba1, Rcor3, Cryl1, Vmn2r29, 2610028H24Rik, Dag1, Itga5*

*D4Mit312*	Adipose tissue	*Gjb3, Tnni2, Tnnc2, Ckm, Pvalb, Lamb3, Slc39a3, Mmp3, Atp2a1, 1110008J03Rik, Mark1, Hipk1, Ptcra, Tcap, Mfap4, 2510006D16Rik, C430004E15Rik, Myh4, Ttn, 9030625N01Rik, A930012M21Rik, 2900011L18Rik, Fntb, Dhx29, Mylpf, Tnnt3, Sphk1, 2410127E18Rik, 3110082I17Rik, Marveld3, 9930104L06Rik, Plekhg4, Flnb, Lpin2*
	Liver	*Vmn2r88, Egr2, Ftsj1, 1810059H22Rik, Frmd5, Gng2, Asxl2, P4ha3, Fpr1, Zfp54, 4921515E04Rik, Rab36, Pou2f1, Syt7, 4930552P12Rik, Klk1b1, Map3k4, Pip5k1b, Rims2*
	Muscle	*Rbm26, Efcab4a, Edg5, Jam2, Ptpn14, OTTMUSG, Hif1a, Cdca7l, Ep400, 1110034A24Rik, Bet1l, Ick, Bcl9, Shc4, Adam12, Col9a1*
	Pancreas	*Gnb1, Pdcd6ip, Stub1, **Ctrc**, Alkbh5, Xpnpep3, Slc25a29, Zswim5, H13, 4930534B04Rik, Ascc2, St3gal6, Rock2, Dnm3os, Bet1l, Lox, Gjb4, Xpc*

*D11Mit41*	Adipose tissue	*Ptpn11*
	Liver	*Dusp3*
	Muscle	*Exoc7, Plekhg1*
	Pancreas	*Pja2*

*D1Mit215*	Adipose tissue	*Isl1, Plekhf2, Baalc, Dusp18, Lim2, A130004G07Rik, Vstm2l*
	Liver	*Rnd3, Slc7a4, Jarid1b, Clasp1, 1700009P17Rik, Casp3, D730045B01Rik, Foxp4, Nono, 5830411G16Rik, 6030458A19Rik, Dok7, Centb1, Ddef2, 5830418K08Rik, Tmem179, Cux1, 1110001A07Rik, Sp6, Zfp560, Pnmal1, Npb, 7420416P09Rik, Vta1, Crybb1, 9930031P18Rik, Tmem202, 4932409I22Rik, Nav2, Bhlhb8, Skiv2l2, Zfp383, Cxcl9, Akr7a5, Ttc3, Itga3, Gpatch2, Ash2l, Dub2a, Gypa, 2210407C18Rik*
	Muscle	*2810454L23Rik, Ugcgl2, 4930558K02Rik, Cercam, Fbxo10, Rbms3, C130039O16Rik, D130051D11Rik, Ifnar2, Smchd1, Edg6, Fgd5, Sertad3, Mcm4*
	Pancreas	*Sept8, Bloc1s3, Mchr1, Mfhas1, Prpf40a, Morc2a, Slc40a1, Haghl, Grb10, 8430437O03Rik, 2210404J11Rik, Fndc4, Hsd17b4, Sf3b2, Alkbh1, Zfp652, Plcxd2, B430119L13Rik, Pdzk1ip1, Epha10, Spnb2, Lrch1, 9930031P18Rik, Mfap5, Det1, Fcrls, Myh11, Ifna5, Gpx6, Zhx3, 4930503L19Rik, Ascc3l1, Gli3, Tubb5, Pex10, Snf1lk, Cog1, Gm693, Pogz, Dbil5, Capn9, Itm2a, Ttc3, Zfp282, Cldn23, Clec4a2, Eif3s10*

*D14Mit107*	Adipose tissue	*Spg11*
	Liver	*Hbb-b1, Gpr146, Hba-a1*
	Muscle	*Shq1*

Genes in bold are putative *cis*-acting transcripts.

As another method to select possible candidate genes, we searched for gene expression corresponding to genes located near the QTLs that were correlated with physiological traits [11]. Gene expression levels of many genes in adipose tissue were correlated (P < 0.05) with body weights and/or fat pad weights (Table 5). Notable ones included insulin receptor substrate 1 (*Irs1*) gene and monoacylglycerol O-acyltrasferase 1 (*Mogat1*) gene located within the Chr 1 obesity QTL interval near *D1Mit215*. Within the Chr 1 QTL interval, insulin-like growth factor binding protein 2 (*Igfbp2*) gene expression levels in liver were also negatively correlated with mesenteric fat pad weights. Interestingly, we also observed increased gene expression levels of multiple chemokine (C-C motif) ligands, including *Ccl9*, *Ccl6*, and *Ccl3 *in adipose tissue, being positively correlated with body and fat pad weights. These genes all map on the Chr 11 body weight QTL interval near *D11Mit41*.

**Table 5:** Correlations of the gene expression levels with physiological traits.

Tissue	Positively correlated	Negatively correlated	Trait
*Near D1Mit113*			
Adipose tissue	*Prrx1, Creg1, Nuf2, Sh2d1b1, Fcgr4, Fcgr3, Fcer1g, Adamts4, Cd84, Pea15a, Atp1a4, Slamf9, Tagln2, Vsig8, Slamf8, Wdr26, Degs1, Kctd3, Atf3*	*Gas5, Opn3, Sdccag8, Hnrnpu, Trp53bp2, Iars2, Ptpn14*	BW FPW
Muscle	*Sft2d2, Dedd*,	*Sdhc* *Mrps14, Prrx1, Nme7, Mpzl1, Cd247, Lrrc52, Fcrla*, *Adamts4, Pvrl4, Cd244, Slamf7, Igsf9, Tagln2*	Chol Tg

*Near D11Mit132*			
Adipose tissue	*Ccl6, Rab5c, Atp6v0a1, Psmc3ip, Tubg1, Psme3, Dusp3, Grn, Nmt1, Plekhm1, Cdc27, Mrc2, Tanc2, Wipi1, Kpna2*	*Stat5b, Rdm1, Rprml, Helz*	BW FPW
Muscle	*Prkar1a, Sgca, Spop, Krtap3-1*	*Fkbp10, Acly, Stat3, Vps25, Cntd1, Psme3, Ifi35, Brca1, Slc25a39, Gpatch8, Gm1564, Eftud2, Hexim2, Lyzl6, Wnt3, Crhr1, Arsg, Slfn10, Slfn3, Kal1, Lhx1, Ggnbp2, Usp32, Ptrh2, Dhx40, Rad51c, **Msi2**, Car10, Mbtd1, Mycbpap, Rsad1, Hils1, Zfp652, Ube2z, Calcoco2, Skap1, Scrn2, Kpnb1, Pip4k2b, Med1, Crkrs, Gsdma2, Wipf2, Krt12*	Tg

*Near D4Mit178*			
Adipose tissue	*Col15a1, Nipsnap3a, Svep1, Slc31a1, Orm1, Tnc, Megf9, Mpdz*	*Rgs3, Orm3*	BW FPW
Muscle		*Mup4, Mup3, Zfp37, Orm2, Pappa, Dbc1, Cdk5rap2, Rasef*	Tg

*Near D8Mit242*			
Adipose tissue	*Tmem208, Lypla3, Cyb5b, Hp, Mlkl, Cenpn, Gcsh, Cotl1, Gins2*	*Cirh1a, Ces3, Bbs2, Terf2ip, Fbxo31*	BW FPW
Muscle	*Cog8, Znrf1, Gse1*	*Cdh8, Cklf, Cmtm2a, **Ccdc79**, Lcat, Prmt7, Sntb2, Adat1, Terf2ip, Mon1b, Jph3, Cdt1, Zfp319, Gins3, Setd6*	Tg

*Near D3Mit304*			
Muscle		***Agtr1b***, *Tbl1xr1, Arpm1, Samd7, Cldn11, Slc7a14, **Pik3ca**, **Ccdc39**, Sox2*	Tg
Adipose tissue	*Fabp5, Sirpb1, Aadacl1, Tpd52*	*Lrrcc1, Car3, Mynn, Phc3, Zfhx4*	BW FPW

*Near D4Mit312*			
Muscle	*Nadk*	*Eif4g3, Ubxd3, Pax7, Arhgef19, Tnfrsf8, Rex2, Clcn6, Fbxo6, Gpr153, Morn1, Gabrd, Ssu72, Ttll10, Dhdds, Lin28, Sepn1, Srrm1, Rcan3, Cnr2, Htr1 d, Ephb2, Ptafr, Eya3*	Tg
Adipose tissue	*Efhd2, Lzic, Pgd, Plod1, Dnajc16, Necap2, Mfap2, Atp13a2, Igsf21, Capzb, Pla2g2e, Hspg2, C1qc, Ephb2, Clic4, Ldlrap1, Paqr7, Stmn1*	*Sdf4, Thap3, Per3, Park7, H6pd, Tmem201, Tardbp, Gale, Srrm1, Syf2, Tmem57*	BW FPW

*Near D11Mit41*			
Adipose tissue	*Eif4a1, Atp6v0a1, Psmc3ip, Dusp3, Nmt1, Mpdu1, Cd68, Garnl4, Serpinf1, Slc43a2, Pitpna, Blmh, Pigs, Evi2a, Rhbdl3, Ccl2, Ccl7, Ccl9, Ccl6, **Ccl3**, Dynll2, Mmd, Lrrc59, Col1a1, Top2a*	*Rdm1, Ssh2, Unc45b, Acaca, Ggnbp2, Med13, Vezf1*, *Tmem100, Spop, Kpnb1, Erbb2, Thra*,	BW FPW
Muscle	*Sgca, Spop, Krtap3-1*	*Fkbp10, Acly, Cntd1, Ifi35, Lyzl6, Aurkb, Trp53, Alox12, Itgae, Phf12, Nek8, **Spag5**, Nos2, Rhot1, Zfp207, Fndc8, Slfn10, Slfn3, Kal1, Lhx1, Ggnbp2, Usp32, Ptrh2, Dhx40, Rad51c, **Msi2**, Car10, Mbtd1, Mycbpap, Rsad1, Hils1, Zfp652, Ube2z, Calcoco2, Skap1, Scrn2, Kpnb1, Pip4k2b, Crkrs, Gsdma2, Wipf2, Krt12*	Tg

*Near D1Mit215*			
Adipose tissue	*Slc11a1, Col6a3*	*Ikzf2, **Usp37**, **Cyp27a1**, **Mogat1**, **Irs1***	BW FPW
Liver		***Igfbp2***	FPW
Muscle		*Sumo1, Spag16, Tnp1, Vil1, Plcd4, Zfp142, Ttll4, Cyp27a1, Des, Epha4, Pax3, Mrpl44, Cops7b, Chrng*	Tg

*Near D14Mit107*			
Adipose tissue		*Dock9*	BW FPW
Muscle		*Dock9, Dzip1, Oxgr1, Ipo5, Farp1, Fgf14*,	Tg

Genes located in physiological trait QTL intervals, showing correlations of the gene expression levels with physiological traits (P < 0.05) are detected by microarray and regression analyses using the subset of F2 mice (n = 16). Genes in bold are confirmed by qRT-PCR. BW, body weight; FPW, fat pad weight; Chol, cholesterol; Tg, triglyceride.

### Real-time quantitative RT-PCR (qRT-PCR)

We conducted qRT-PCR analysis for candidate genes obtained from the microarray data or literature search (Table 6). Gene expression levels of *Ccdc46 *in liver, *Ctrc *in pancreas, and *Sirpb1 *in adipose tissue were significantly up-regulated in TH mice compared with B6 mice. On the other hand, the level of gene expression of *1700009P17Rik *was significantly down-regulated in liver from TH mice compared with B6 mice. The gene expression levels of *Irs1*, *Mogat1*, and *Igfbp2 *were significantly down-regulated in adipose tissue from TH mice compared with B6 mice.

**Table 6:** Real-time quantitative RT-PCR for selected genes in B6 and TH mice (males, 16 week, n = 5 each group).

Gene symbol	Gene name	Chr	Near marker	Trait	Tissue	Fold	*P*
*1700009P17Rik*	RIKEN cDNA 1700009P17 gene	1	*D1Mit113*	Tg Chol	Liver	0.2	0.0007
*Ccdc46*	Coiled-coil domain containing 46	11	*D11Mit132*	Tg	Liver	7	0.0019
*Sirpb1a*	Signal-regulatory protein beta 1A	3	*D3Mit304*	Chol	Adipose tissue	2.6	0.02
*Ctrc*	Chymotrypsin C (caldecrin)	4	*D4Mit312*	Glu	Pancreas	17	<0.0001
*Igfbp2*	Insulin-like growth factor binding protein 2	1	*D1Mit215*	FPW	Liver	0.50	0.05
					Adipose tissue	0.1	<0.0001
*Cyp27a1*	Cytochrome P450, family 27, subfamily a, polypeptide 1	1	*D1Mit215*	BW FPW	Adipose tissue	1.2	0.10
*Irs1*	Insulin receptor substrate 1	1	*D1Mit215*	BW FPW	Adipose tissue	0.6	0.05
*Mogat1*	Monoacylglycerol O-acyltransferase 1	1	*D1Mit215*	FPW	Adipose tissue	0.2	0.0002
*Usp37*	Ubiquitin specific peptidase 37	1	*D1Mit215*	BW FPW	Adipose tissue	1.0	0.90
*Spag5*	Sperm associated antigen 5	11	*D11Mit41*	Tg	Muscle	1.02	0.95
*Ccl9*	Chemokine (C-C motif) ligand 9	11	*D11Mit41*	BW FPW	Adipose tissue	2.2	0.01
*Ccl6*	Chemokine (C-C motif) ligand 6	11	*D11Mit41*	BW FPW	Adipose tissue	1.4	0.20
*Ccl3*	Chemokine (C-C motif) ligand 3	11	*D11Mit41*	BW FPW	Adipose tissue	5.5	<0.0001
*Msi2*	Musashi homolog 2 (Drosophila)	11	*D11Mit41*	Tg	Muscle	1.03	0.75
*Agtr1b*	Angiotensin II receptor, type 1b	3	*D3Mit304*	Tg	Muscle	1.11	0.85
*Pik3ca*	Phosphatidylinositol 3-kinase, catalytic, alpha polypeptide	3	*D3Mit304*	Tg	Muscle	0.91	0.24
*Ccdc39*	Coiled-coil domain containing 39	3	*D3Mit304*	Tg	Muscle	0.66	0.40
*Ccdc79*	Coiled-coil domain containing 79	8	*D8Mit242*	Tg	Muscle	0.59	0.40
*Apoa2*	Apolipoprotein A-II	1	*D1Mit113*	Tg Chol	Liver	1.07	0.08
*Insig2*	Insulin induced gene 2	1	*D1Mit113*	Tg Chol	Muscle	0.95	0.73
*Zfp69*	Zinc finger protein 69	4	*D4Mit312*	Glu	Adipose tissue	2.2	0.03

Chr, chromosome; Fold, the fold change in TH vs. B6 (TH/B6); Tg, triglyceride; Chol, cholesterol; Glu, glucose; FPW, fat pad weight; BW, body weight.

### Apolipoprotein A-II (*Apoa2*) gene in dyslipidemia in TH mice

**Figure 5:**
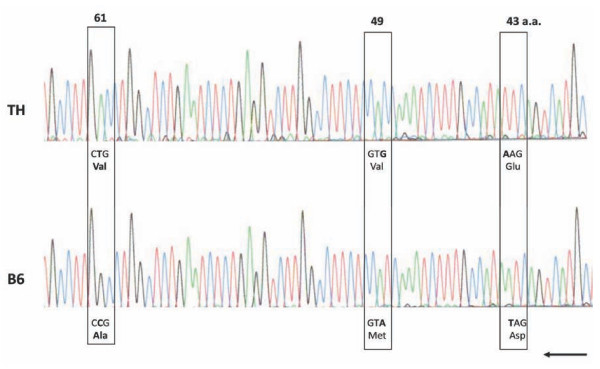
***Apoa2 *cDNA sequence comparison in TH and B6 strains**.

*Apoa2 *gene maps to the distal region of mouse Chr 1, near the Chr 1 QTL linked to hypercholesterolemia and hypertriglyceridemia in the F2 mice. Through transgenic and knockout studies it has been shown that *Apoa2 *is involved in controlling plasma cholesterol and triglyceride levels and over-expression causes insulin resistance and obesity [12-14]. In order to test if *Apoa2 *could be the Chr 1 hyperlipidemia QTL, we conducted sequence comparison of the coding region between TH and B6 mice. There were 7 nucleotide substitutions in the *Apoa2 *coding sequence, resulting in 3 amino acid differences in the two strains (Figure 5). The gene expression level of *Apoa2 *was not significantly different between TH and B6 mice in liver (Table 6).

## Discussion

Through genome-wide linkage scans using an F2 intercross population from TH and B6 strains, we identified multiple QTLs and interacting loci linked to hyperlipidemia, hyperglycemia, and obesity phenotypes. Further, genetic study of gene expression in a subset of this F2 population led to potential candidate genes for the QTLs.

Some limitations of this study need to be recognized. First, marker spacing in this study was approximately 20-cM, which was at the lower end of the generally accepted marker density (10 - 25 cM) for QTL genome scans [15] and could cause underestimation of the number of QTLs identified in this study. However, it has been shown that the power of detecting a QTL is barely influenced by marker spacing in the range of 10 to 20 cM, with most (over 70%) of the power retained even at 50 cM spacing [16]. Therefore, it is unlikely that there are QTLs with stronger effect than were identified in our F2 cross. Fine mapping studies to further define the QTLs identified will provide further validation. Second, measurements of plasma glucose, lipid and insulin levels are sensitive to multiple environmental perturbations, such as animal husbandry, testing procedures, and environmental conditions. This creates phenotypic variation that may result in less power to detect the genetic determinants for physiological differences or inconsistency in detected QTLs across time. This may have caused the observed fluctuations in QTL locations over time. The third potential limitation was running microarray analysis on a small subset of F2 mice selected from the upper and lower tails of the frequency distribution of the plasma triglyceride levels without severe hyperglycemia. Populations with phenotypic extremes are known to be most genetically informative as genes influencing to the disease should be concentrated in the extremes of the populations. Thus, even with small number of mice, our data from multiple tissues did identify potential novel candidate genes that may be involved in pathogenic mechanisms in TH mice.

In mice, *Apoa2^b ^*allele, characterized by Ala61-to-Val61 substitution, has been reported to be hypermorphic in increasing plasma cholesterol levels and appears in multiple mouse strains [17,18]. The same polymorphism of *Apoa2 *gene was revealed in TH strain, suggesting *Apoa2 *as the candidate gene underlying the Chr 1 QTL linked to hypercholesterolemia. Indeed, many cholesterol QTLs were previously detected in the *Apoa2 *region in separate genetic crosses of mice and *Apoa2 *was suggested as a likely candidate across the strains [19]. Functional studies related to *Apoa2^b ^*allele in TH mice are warranted.

Unlike cholesterol, age-specific genetic loci were found for plasma triglyceride levels, suggesting that different genetic mechanisms are responsible for the onset versus progression of hypertriglyceridemia in TH mice. It is noteworthy that the Chr 1 locus linked to hypertriglyceridemia at 8 weeks of age overlapped the Chr 1 hypercholesterolemia QTL. It might be speculated that the onset of both hypertriglyceridemia and hypercholesterolemia shares the same genetic factors, but distinct genetic mechanisms are involved in the progression of the disease in TH mice. This age-related genetic effect has been previously recognized and appreciated in complex traits including diabetes and obesity [20,21]. Data collection at multiple time points provides power to detect age-dependent effects that can be easily missed with a single time point study.

The Chr 11 and Chr 4 hypertriglyceridemia QTLs map near the loci previously observed for atherosclerosis in mice; *Ath19 *on Chr 11 [22,23] and *Ath8 *on Chr 4 [22,24]. The authors proposed the angiopoeitin-like protein 3 (*Angptl3*) gene, containing a coiled-coil domain, as a positional candidate gene for the *Ath8 *[24]. Interestingly, *Angptl3*-null mice show markedly low plasma triglyceride concentrations [25]. The Chr 8 hypertriglyceridemia QTL maps to the region of *Tgl1 *linked to serum triglyceride levels in KK/Ta × (BALB/c × KK/Ta) F1 backcross mice [26].

A major QTL for the hyperglycemic (diabetic) trait was identified on the distal region of Chr 4. Previously, several QTLs for diabetes and diabetes-related phenotypes have been mapped in this region in independent cohorts of mice and rats [27]. Among those, QTLs directly linked to plasma glucose levels included *Nidd1 *in F2 mice from NZO × NON, a locus near *D4Mit203 *in F2 mice from C57BL/KsJ × DBA/2, and *Nidd/SJL *in backcross mice from (NZO × SJL) × NZO.

Recently, the zinc finger protein 69 (*Zfp69*) gene was identified as a candidate for the diabetes QTL of *Nidd/SJL *[28]. An allelic variation of *Zfp69 *was observed in multiple inbred strains; allele carried by B6 and NZO strains causing truncated mRNA was associated with reduced diabetes susceptibility, while allele carried by SJL and NON strains producing normal mRNA was diabetogenic [28]. It is possible that the TH strain, which is close to Swiss family strains http://jaxmice.jax.org/strain/005314.html, may carry the SJL allele of *Zfp69*. This notion is possibly supported by the observed higher gene expression of *Zfp69 *in TH mice compared to B6 mice (Table 6).

Body weight is a compound trait reflecting the weights of lean muscle and bones as well as fat mass. A QTL near *D11Mit41 *on Chr 11 was very significantly linked to body weight (Table 2). This locus was also associated with carcass weight, suggesting its major effect on lean mass. Among multiple body weight-related QTLs mapped to this Chr 11 interval [29], the *Wg4 *(also known as *Q11Ucd1*) locus behaves similarly to the Chr 11 body weight QTL, affecting growth rate and carcass lean mass in *hg/hg *F2 population from a cross of B6-*hg*/*hg *x CAST/EiJ [30].

A major QTL near *D1Mit215 *on Chr 1 was linked to all the fat pad weights and body weights at 24 weeks of age. This chromosomal interval contains *Nob3*, significantly linked to adiposity (defined as body weight and body fat) in F2 progeny from NZO and B6 mice [31]. Several other QTLs associated with body weights were identified in this interval in mice, namely *W3q12 *and *W10q6 *[32] and *Bwtq1 *[33]. The human orthologous region of the Chr 1 QTL is 2q33-37. Based on known functional relationship to metabolism, a few genes mapped in this region, including *Irs1*, *Mogat1 *and *Igfbp2*, are considered as candidates.

Obesity has been known to be a low-grade chronic inflammatory disease [34]. In this study, we observed that the gene expression levels of multiple chemokines, including *Ccl9 *and *Ccl3*, were significantly increased in adipose tissue of TH mice compared to B6 mice. The gene expression levels of these genes were also positively correlated with body and fat pad weights in the F2 mice (Table 5).

Previous research found genetic determinants of diabetes and obesity in TH mice in backcross population from F1(B6 × TH) × TH, including diabetes QTLs on Chr 19 (*Tanidd1*) and Chr 13 (*Tanidd2*) and obesity QTLs on Chr 7 (*Tabw*), Chr 4 (*Tafat*) and Chr 6 (*Tabw2*) [5,35]. Two loci on Chr 18 and 16 interacted with *Tanidd1 *and *Tanidd2*, respectively. These QTLs were not detected in the present F2 study. This discrepancy is not totally surprising. As the detection of a QTL is subject to the magnitude of phenotypic variation within genotypes, QTL results in an F2 and a backcross population from the same progenitors can be different [36]. For example, when complete dominance exists for the TH allele of a QTL, the power of a backcross [F1(B6 × TH) × TH] for detecting this QTL is zero. Similarly, QTL interaction effects could be affected by the choice of an F2 or backcross, in part, due to the absence of homozygous mice for B6 alleles in the backcross. Another source of the discrepancy in results from the two studies could be the different physiological conditions of the mice. Non-fasting plasma glucose levels were measured in the backcross study, while 4-hour fasting plasma glucose levels were used in the present F2 study. Therefore, the present findings using F2 population should be taken as additional genetic information underlying the pathogenic mechanisms in TH mice, not just an independent replication.

## Conclusions

In summary, using ~380 male F2 mice from the B6 × TH intercross we detected 12 significant QTLs; 4 for hypertriglyceridemia, 2 for hypercholesterolemia, 1 for hyperglycemia, 2 for body weights, 1 for fat pad weights, and 2 for carcass weights. Polymorphisms in *Apoa2 *gene are suggested to be responsible for the Chr 1 QTL linked to hypercholesterolemia and hypertriglyceridemia. Gene expression analysis added novel aspects and aided the selection of candidates and biological mechanisms for the QTLs. Future studies to define the molecular bases of these QTLs will improve the understanding of genetic contributions in diabetes related syndrome in TH mice and ultimately in humans.

## Methods

### Animals

Mice were maintained on standard rodent chow with 4% fat [Harlan Teklad Rodent Diet (W) 8604, Madison, WI] ad libitum with free access to water (HCl acidified, pH 2.8-3.2) under controlled temperature and humidity with a 12-hour light and dark cycle. All animal studies were carried out with the approvals of The University of Tennessee Animal Care and Use Committee and Marshall University Animal Care and Use Committee. Mice were euthanized by CO_2 _asphyxiation.

### Genetic crosses

Male TH mice were mated to female B6 mice. The resulting F1 hybrid mice were interbred to produce an F2 population. Male F2 mice were fasted for 4 hours at the beginning of the light cycle (6:00-7:00 AM) and blood was collected via the retro-orbital plexus using a heparinized microcapillary tube at 8, 12, 16, 20, and 24 weeks of age. Plasma was obtained by centrifugation (1,200 *g*) at 4°C and plasma levels of glucose, true triglyceride, total cholesterol, and insulin and body weights were measured. At the end of the study, mice were killed and tissues including liver, skeletal muscle, pancreas, and adipose tissue (inguinal, epididymal, mesenteric, retroperitoneal including perirenal, and subscapular fat pads) were collected, frozen in liquid nitrogen, and stored at -80°C for RNA isolation. Respective fat pad weight and carcass weight (body weight without the five fat pads) were also recorded during the dissection.

### Plasma glucose, triglyceride, total cholesterol, and insulin levels

Plasma levels of glucose (TR15103/1530-500, Thermo Electron, Louisville, CO), total cholesterol (TR13421, Thermo Electron, Louisville, CO), and free and total glycerol (337, Sigma, St. Louis, MO) were determined using commercial colorimetric assays. Plasma true triglyceride concentrations were estimated by subtraction of free glycerol from total glycerol. Plasma insulin levels were determined using RIA (RI-13K, Linco Research, St. Charles, MO).

### Genotyping by PCR

Genomic DNA was extracted from tail tips using proteinase K [37] and two series of salt precipitation steps. The DNA was PCR amplified using SSLP primers (Additional file 1 Table S1) purchased (Invitrogen, Carlsbad, CA) or synthesized (Sigma) based on sequences from Mouse Genome Informatics http://www.informatics.jax.org/javawi2/servlet/WIFetch?page=markerQF. The thermal cycle consisted of 95°C for 2 min, followed by 49 cycles of 94°C (20 sec), 50°C (30 sec) and 72°C (40 sec) and a final extension at 72°C (7 min). Amplified products were electrophoretically separated on 3% metaphor (50184, FMC, Rockland, ME)/1% agarose (0710-500G, Amresco, Solon, OH) gels in 0.5 × tris-borate-EDTA buffer, pH 7.4. The DNA was visualized by ethidium bromide (E-1510, Sigma) staining.

### RNA isolation

Total RNA was isolated from liver, muscle (combined soleus and gastrocnemius), pancreas, and adipose tissue (combined inguinal, epididymal, retroperitoneal, perirenal, and subscapular fat pads) using RNeasy Lipid Tissue Midi Kit (75842, QIAGEN, Valencia, CA) according to the manufacturer's instructions. For adipose tissue, muscle and pancreas, the entire tissue was homogenized and total RNA extracted, whereas approximately 50% of the liver was homogenized. Total RNA was further purified using RNeasy MinElute Cleanup Kit (74204, QIAGEN) for microarray analysis.

### Microarray Analysis

Hybridizations were performed at the University of Tennessee Affymetrix Facility (Knoxville, TN) using Affymetrix GeneChip^® ^Mouse Genome 430 2.0 Array (Affymetrix, Santa Clara, CA) following the standard protocol. The Mouse Genome 430 2.0 Array contains 45,000 probe sets on a single array to analyze the expression level of over 39,000 transcripts and variants from over 34,000 well-characterized mouse genes (Affymetrix). Total RNA isolated from adipose tissue, liver, muscle, and pancreas of a subset of F2 mice (n = 16) were used for microarray analysis, requiring 64 arrays. The 16 mice were chosen from the upper and lower tails (8 each) for plasma triglyceride distribution of all the male F2 mice, excluding overtly diabetic mice. The phenotypic values of these mice are presented in Table 1.

### Real-time quantitative RT-PCR (qRT-PCR)

Total RNA was isolated from adipose tissue, liver, muscle, and pancreas of B6 and TH male mice at 16 weeks of age as described above. Total RNA (2 μg) was reverse-transcribed with SUPERSCRIPT RT (11904-018, Invitrogen) using oligo d(T)12-18 (18418-012, Invitrogen) as primer to synthesize first-strand cDNA in 20-μl volume according to manufacturer's instructions. The primers used for the real-time PCR were proprietary for *Ctrc *(PPM39932A), *1700009P17Rik *(PPM27997A), *Sirpb1a *(PPM39258A), *Irs1 *(PPM05117E), *Cyp27a1 *(PPM30124A), *Mogat1 *(PPM28787E), *Usp37 *(PPM60128A), *Zfp69 *(PPM40990E), *Insig2 *(PPM26980A), *Pik3ca *(PPM05112A), *Agtr1b *(PPM31347B), *Ccdc79 *(PPM35025A), *Ccdc39 *(PPM28741A), and *Apoa2 *(PPM05347B) (SABiosciences, Frederick, MD). Oligonucleotide primers were also synthesized (Sigma) using sequences obtained from Primer Bank http://pga.mgh.harvard.edu/primerbank for *Ccdc46*, *Igfbp2*, *Ccl3*, *Ccl9*, *Ccl6*, *Msi2*, and *Spag5 *and from the published literature for *36B4 *[38] (Table 7).

**Table 7:** Primer sequences for real-time quantitative RT-PCR.

Gene	Forward Primer (5' - 3')	Reverse Primer (5' - 3')
*Ccdc46*	GCTGAGACCGAGATGACTCTG	GCACTTCGCACCTGATGAGA
*Igfbp2*	CAGACGCTACGCTGCTATCC	CCCTCAGAGTGGTCGTCATCA
*Ccl3*	TTCTCTGTACCATGACACTCTGC	CGTGGAATCTTCCGGCTGTAG
*Ccl9*	CCCTCTCCTTCCTCATTCTTACA	AGTCTTGAAAGCCCATGTGAAA
*Ccl6*	GCTGGCCTCATACAAGAAATGG	GCTTAGGCACCTCTGAACTCTC
*Msi2*	GACCTGTCGCCGATCTCTAC	GCGCTTATGTAATTCCCCACTC
*Spag5*	ACAGTGAGTCTGAGTTCTGCC	CTGTGAGTTTCTTGGTGAGTTCT
*36B4*	GAGGAATCAGATGAGGATATGGGA	AAGCAGGCTGACTTGGTTGC

The real-time PCR reaction was carried out in a 25-μl volume in 1× SYBR Green PCR core reagents (PA-112, SABiosciences) containing 1 μl cDNA template diluate (1:5, v/v) and 6 pmol primers using ABI Prism 7700 or 7300 sequence detection system (Applied Biosystems, Foster City, CA). For each sample, triplicate amplifications were performed and the average measurements used for data analysis.

### RT-PCR and sequencing of the *Apoa2* gene

Total RNA was extracted from pancreas of B6 and TH mice and the RNA (10 μg) was reverse-transcribed as described above. The single-strand cDNA was diluted with water (1:5, v/v), and 2 μl of the diluate was used to amplify *Apoa2 *cDNA using the Expand Long Template PCR System (Roche, Indianapolis, IN). Full length coding sequence was amplified using *Apoa2 *specific primers F1 (5'-AGAATCGCAGCACTGTTCC-3') and R1 (5'-GGAGAAAACAGGCAGAAGG-3') derived from Mus Musculus *Apoa2 *gene mRNA sequences (NM_013474). PCR products were first electrophoresed on a 1.2 % agarose gel. Bands of interest were excised from the gel, and DNA fragments were isolated (K3051-2, Clontech, Palo Alto, CA). The gel-purified PCR products were directly sequenced with primers originally used to amplify the PCR products. Sequencing was carried out automatically with fluorescent tags (3100 Genetic Analyzers, Applied Biosystems).

### Statistical Analysis

#### QTL mapping

QTL analysis was run using R/QTL [39] with composite interval mapping at default settings. Traits that showed positive skew were log transformed. The estimated map showed expansion compared to published marker locations, so the estimated map was used. This had the benefit of resolving putative genotyping errors on Chr 4. Genome wide significance thresholds were established by identifying the 10th (suggestive), 5th (significant) and 1st (very significant) percentiles from 10,000 permutations.

#### Microarray data analysis

Individual probe data were extracted using Bioconductor http://www.bioconductor.org, and the gcRMA (robust multi-array) process used to produce a signal measure for each gene. Statistical analysis was performed using SAS software (Cary, NC). A mixed ANOVA model [40] was run on the normalized data, fitting genotype and tissue treatment effects, and using array variation as the experimental error. Genes with significant (P < 0.05) ANOVA interaction, and significant pair-wise False Discovery Rate [41] were considered differentially expressed. Microarray expression data were also tested for relationship to phenotypes using correlation analysis in SAS [11]. A total of 45,000 probes * 39 phenotypes = 1.75 million regressions were run for each tissue. Similarly, analysis of variance in SAS was conducted to identify associations between markers and gene expression in each tissue, comparing marker genotypes for differences in mean expression at all microarray probes. P-values from both analyses were protected at a 5% False Discovery Rate. Microarray data have been submitted to the gene expression omnibus http://www.ncbi.nlm.nih.gov/geo/ under the accession number GSE24637.

#### Real-time qRT-PCR data analysis

Triplicate threshold cycle times were averaged for each mouse, and strain differences tested using a one-way ANOVA in SAS software (Cary, NC), with the 36B4 control gene values as a covariate to avoid analysis of ratios. Data are presented as relative fold-change using B6 mice as the reference by 2^(ΔCt of TH mice - ΔCt of B6 mice) ^[42], where ΔCt represents the treatment mean from ANOVA.

#### Physiological data analysis

Data analysis was conducted by ANOVA with StatView 5.0 (Abacus Concepts, Berkeley, CA). Differences were considered significant at *P *< 0.05. All data are presented as mean ± SEM.

## Authors' contributions

TPS did phenotyping and genotyping of F2 mice and was in charge for all aspects of animal care. HYK did microarray analysis. AMS conducted statistical analysis of the genetic and genomic data. JHK conceived the study and was primarily responsible for its coordination and design and did qRT-PCR and sequencing analysis. AMS and JHK drafted the manuscript, tables and figures. All authors read and approved the final manuscript.

## Supplementary Material

Additional file 1 **Table S1 - Simple sequence length polymorphic markers for the genome-wide scans of the (B6 × TH) F2 mice**. The file contains the list of genetic markers and their estimated genetic map positions used in this study. Click here for file
