# Supplementary material for: Genetic and genomic analysis of hyperlipidemia, obesity and diabetes using (C57BL/6J × TALLYHO/JngJ) F2 mice
Source: BMC Genomics. 2010 Dec 19;11:713. doi: 10.1186/1471-2164-11-713 (PMC3022919; doi:10.1186/1471-2164-11-713)
Supplement: Additional file 1 — Table S1 - Simple sequence length polymorphic markers for the genome-wide scans of the (B6 × TH) F2 mice. The file contains the list of genetic markers and their estimated genetic map positions used in this study. [file 1471-2164-11-713-S1.PDF]

Additional file 1.

Table S1 - Simple sequence length polymorphic markers for the genome-wide scans of the (B6 x TH) F2 mice.

Marker	Chromosome	Position (cM)	Marker	Chromosome	Position (cM)
D1Mit213	1	25.70000	*D9Mit142*	9	35.62135
D1Mit215	1	39.09944	*D9MIt212*	9	82.23472
D1Mit26	1	54.02135	*D10Mit80*	10	4.00000
D1Mit113	1	92.56623	*D10Mit130*	10	40.45787
D1Mit206	1	96.92949	*D10Mit11*	10	74.11835
D2Mit1	2	1.00000	*D11Mit20*	11	20.00000
D2Mit296	2	29.57183	*D11Mit86*	11	37.55040
D2Mit56	2	53.53994	*D11Mit41*	11	69.06976
D2Mit17	2	86.53566	*D11Mit132*	11	82.01853
D2Mit22	2	103.63717	*D12Mit83*	12	6.00000
D2Mit200	2	138.33009	*D12Mit34*	12	27.01363
D3Mit304	3	5.60000	*D12Mit233*	12	66.42436
D3Mit40	3	42.68755	*D13Mit205*	13	5.00000
D3Mit106	3	64.88324	*D13Mit26*	13	59.21726
D4Mit97	4	6.30000	*D13Mit148*	13	95.52672
D4Mit178	4	35.50063	*D13Mit35*	13	132.74869
D4Mit37	4	61.37653	*D14Mit53*	14	12.5000
D4Mit312	4	97.34623	*D14Mit102*	14	36.7962
D4Mit42	4	105.47051	*D14Mit107*	14	76.0684
D4Mit208	4	110.14027	*D15Mit174*	15	6.70000
D5Mit193	5	1.00000	*D15Nds2*	15	23.19098
D5Mit80	5	21.06078	*D15Mit2*	15	71.02499
D5Mit20	5	58.25388	*D16Mit181*	16	4.30000
D5Mit101	5	132.21915	*D16Mit4*	16	29.14245
D6Mit93	6	26.29000	*D16Mit152*	16	71.19056
D6Mit29	6	44.26399	*D17Mit143*	17	5.00000
D6Mit339	6	80.85577	*D17Mit54*	17	50.59464
D7Mit306	7	1.70000	*D17Mit123*	17	96.88107
D7Mit231	7	38.40287	*D18Mit68*	18	11.00000
D7Mit109	7	111.37320	*D18Mit53*	18	39.33691
D8Mit95	8	8.00000	*D18Mit47*	18	74.83130
D8Mit339	8	27.79628	*D19Mit30*	19	20.00000
D8Mit242	8	55.75491	*D19Mit66*	19	42.58484
D9Mit323	9	6.00000	*D19Mit71*	19	69.08206
